# Systematic review of clinical practice guidelines for the management of neovascular age-related macular degeneration

**DOI:** 10.1038/s41433-025-03829-8

**Published:** 2025-05-15

**Authors:** Jennifer I. Lim, Stella Ko, Mark McAllister, Nancy Faux, Komal Bawa, Elizabeth Mearns, Shriji Patel, Galin Spicer, Amanda Martinez, David Tabano

**Affiliations:** 1 Department of Ophthalmology and Visual Sciences, University of Illinois Chicago, Chicago, IL USA; 2 Genentech, Inc., South San Francisco, CA, USA; 3 College of Medicine, The Ohio State University, Columbus, OH, USA

**Keywords:** Diagnosis, Eye diseases

## Abstract

**Background/Objectives:**

To assess geographically global clinical practice guidelines (CPGs) for neovascular age-related macular degeneration (nAMD) management.

**Methods:**

A systematic literature review (SLR) of CPGs for nAMD management was conducted using Embase and MEDLINE databases, Guideline Central, Health Technology Assessment bodies, professional ophthalmology associations, and backwards citation tracking. CPGs published between January 2010–October 2023 were included and independently assessed by four reviewers using the Appraisal of Guidelines for Research and Evaluation II (AGREE II). CPGs were qualitatively assessed for anatomical measurements (optical coherence tomography [OCT] and visual acuity [VA]). PROSPERO identification is CRD42023473223.

**Results:**

Nine of 147 identified global CPGs were included in the SLR for diagnosis, treatment, and disease monitoring for nAMD. Overall AGREE II scores were 62–95 (mean [standard deviation] score 75 [10.6]). Strongest domains were Scope and Purpose (86.6 [11.0]), Clarity of Presentation (84.3 [13.0]), and Editorial Independence (89.1 [15.4]); Stakeholder Involvement (63.4 [16.6]), Applicability (73.0 [12.6]), and Rigor of Development (55.4 [25.9]) were lowest. 4/9 CPGs were “Recommended” by reviewers, and 5/9 were “Recommended with Modifications”. All CPGs recommended OCT for initial diagnosis. 2/9 CPGs did not mention VA. For managing pharmacological interventions, 4/9 CPGs recommended using VA, and three recommended OCT. Eight CPGs recommended using either VA or OCT for disease monitoring while on anti-vascular endothelial growth factor (VEGF) treatment. 6/9 CPGs recommended screening for VA and 7/9 CPGs recommended using OCT to change anti-VEGF intervals.

**Conclusion:**

CPG methods, recommendations on applicability in resource-constrained systems, and patient advocacy/perspectives will improve CPG trustworthiness and transparency.

## Introduction

Age-related macular degeneration (AMD) continues to be a leading cause of vision loss and blindness in people >55 years of age, accounting for about 6–9% of patients with blindness globally within this demographic [1]. The worldwide prevalence of AMD is estimated to increase to approximately 288 million (credible interval, 205–399) by 2040 [2]. This increase in AMD is likely to decrease the quality of life for the elderly and increase the economic/social burden for health care providers worldwide [3]. Neovascular AMD (nAMD) develops when blood vessels grow into the macula and if untreated lead to scarring and central vision loss. Although intravitreal injections of anti-vascular endothelial growth factor (VEGF) agents have changed the disease trajectory and become standard of care [4], variability in clinical practice exists across geographic regions [5].

Clinical practice guidelines (CPGs), often developed by professional societies, are instrumental in delivering disease-specific treatment guidance and recommendations to healthcare providers to streamline patient care and enhance outcomes [6]. However, CPGs often vary in quality [7]. Therefore, to improve the comprehensiveness and transparency of reporting, the Appraisal of Guidelines for Research and Evaluation II (AGREE II) instrument was developed to evaluate CPG quality by systematically assessing the strengths and weaknesses, especially of the methodological rigour [8–10].

Diagnosis of nAMD includes a thorough history of the patient’s visual symptoms as well as an ophthalmic examination, including measurement of visual acuity (VA) anterior segment and dilated ophthalmoscopy, optical coherence tomography (OCT), and fundus fluorescein angiography, when indicated [11]. OCT is of particular importance when diagnosing and monitoring nAMD because it is a non-invasive objective retinal imaging test that allows retina specialists to track changes in the retinal thickness that are characteristic for nAMD [12], as well as an important biomarker for nAMD disease activity [13]. The majority of nAMD randomised controlled trials assessing anti-VEGF agents historically used VA as the primary endpoint [14–16], but published research has shown that there may be limited correlation between optical coherence tomography (OCT) and VA [17]. Indeed, OCT is leveraged as the primary measure of disease activity to support further treatment decisions in nAMD disease management [18,19]. Therefore, it is important to improve our understanding of how retina specialists interpret these measures and how they are used in clinical practice. As such, obtaining a comprehensive understanding of how explicitly the CPGs support the use of these assessments in the care continuum would be helpful to minimise the inequality of patient care.

We sought to assess the quality of global CPGs using AGREE II to provide direction on the development of future rigorous guidelines and to qualitatively assess recommendations for anatomical outcomes using OCT and VA measures. With the evolving ophthalmological treatment landscape, there is a growing need to clarify management recommendations that guide practitioners to ensure patient care is optimised to minimise inconsistencies. To our knowledge, no previous systematic literature review (SLR) of CPGs for the management of nAMD has been published. The primary aim of this SLR was to identify, summarise, and critically review the diagnostic methods and treatment strategies recommended in published nAMD CPGs from various countries.

## Methods

### Eligibility criteria for considering studies for this review

#### Search methods for identifying studies

Search strategies were developed using a combination of keywords and controlled vocabulary with filters for English-language only CPGs published after 2010. Searches were conducted in MEDLINE and Embase databases to identify CPGs published between 1 January 2010 and 20 October 2023. A full list of the search terms is detailed in Supplementary Table S1. Manual backwards citation tracking of references from included CPGs and review articles was performed to identify as many additional relevant CPGs in the grey literature that may have not been identified in the database searches (e.g., EyeWiki, Guideline Central, Google Scholar, and ophthalmology and retina society portals [e.g., American Academy of Ophthalmology, International Council of Ophthalmology, American Society of Retina Specialists, The Royal College of Ophthalmologists, and European Society of Retina Specialists]).

#### Eligibility criteria for considering studies for this review

Eligible CPGs from any country that recommended management strategies for adult patients with AMD were included. Country adaptations of CPGs or those limited to a single anti-VEGF agent were considered outside the scope of this literature review and were excluded.

Clinical expert consensus statements were not included as these lack a detailed method describing how recommendations were made, whereas CPGs are evidence-based and provide a rationale describing the studies and evidence supporting a particular recommendation.

### Study selection

Two reviewers independently screened titles and abstracts (S.K. and E.M.) to identify publications that met the inclusion criteria. Where possible, discrepancies were resolved between the two reviewers by discussion; however, if an agreement was not reached, a third reviewer (K.B.) resolved any disagreements.

### Data collection and risk of bias assessment

A standardised extraction template in Google Forms^®^ was developed to capture and present key evidence from each CPG included. Two independent reviewers (E.M. and N.F.) extracted the following data from each CPG: authors, title, year of publication, professional society, country/region, funding, medical specialty of CPG development committee, recommendations regarding the use of OCT and VA in the diagnosis, treatment, and management of nAMD. Consensus methods utilised by the CPG were also captured (e.g., anonymous voting such as the Delphi method, an informal consensus, GRADE). The Delphi method obtains a wider range of opinions because of the anonymous feedback preventing group members from conforming to the opinion of others [20].

### Data synthesis, reporting quality, and analysis

Results of the SLR were summarised qualitatively using narrative synthesis. If an agreement was not reached, a third reviewer (K.B. or D.T.) resolved any disagreements.

The reporting quality of CPGs that were included was independently assessed by four reviewers (E.M., N.F., M.M., S.K.) using the validated AGREE II instrument. AGREE II is composed of 23 items across six quality domains: Scope and Purpose, Stakeholder Involvement, Rigor of Development, Clarity of Presentation, Applicability, and Editorial Independence [10] (Supplementary Table S2). All reviewers were trained on the AGREE II tool before completing the appraisal. All reviewers rated each item on a 7-point scale from 1 (strongly disagree) to 7 (strongly agree). After each reviewer independently scored the CPGs, domain percentages were calculated using the AGREE II methodology (domain scores were calculated by summing up all the scores of the individual items in a domain and by scaling the total as a percentage of the maximum possible score for that domain).

Since the AGREE II tool does not provide a threshold percentage score to dictate level of quality in each domain, nor was a consistent method used in the literature, the reviewers instituted the following threshold: low quality (0–30%), moderate (31–70%), and high (≥71%) quality.

An overall assessment of the CPG included two items: overall quality rating of the CPG and whether the CPG would be recommended for use in practice using a 3-point scale (recommend, recommend with modifications, do not recommend). Overall quality rating percentages were calculated by summing up scores across all four reviewers and scaling across the total maximum possible score across all domains. For the overall recommendation for use in clinical practice, independent reviewers used their judgement as to the quality of the CPG, taking into account the criteria in the AGREE II assessment process. Results of the review were summarised qualitatively using narrative synthesis.

This publication followed the methodology of the Preferred Reporting Items for Systematic Reviews and Meta-analyses Reporting (PRISMA) guidelines 2.0 [21]. The PROSPERO identification for this SLR is CRD42023473223.

## Results

### General overview of included guidelines

**Fig. 1:**
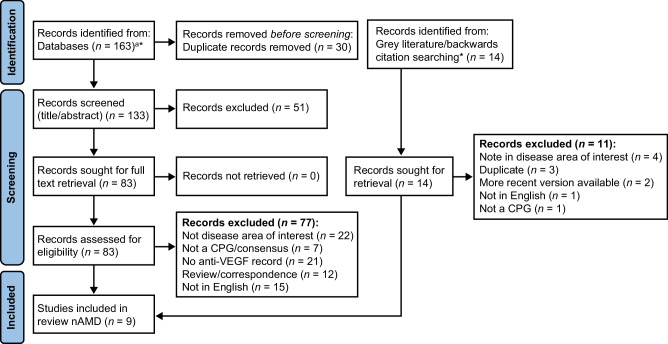
PRISMA flow diagram including database searches and grey literature sources [21]. ^a^Initial literature search identified publications in DMO, nAMD, DR, and RVO. For more information, visit: http://www.prisma-statement.org/; *Two CPGs made recommendations on both DR and DMO. CPG clinical practice guideline, DMO diabetic macular oedema, DR diabetic retinopathy, nAMD neovascular age-related macular degeneration, RVO retinal vein occlusion, VEGF vascular endothelial growth factor.

A total of 163 publications were identified through the literature search and an additional 14 were captured during a search of the grey literature and backwards citation tracking (total 177; Fig. 1). After removing 30 duplicates, 147 CPGs underwent a title and abstract screen with 83 full text and 14 grey literature records subsequently reviewed. After being reviewed based on inclusion and exclusion criteria, nine CPGs for nAMD were included in the study (Table 1). These CPGs were published between 2012 to 2022. Geographically, the CPGs represented a diversity of regions, with two from North America, four from Europe, and three from the Asia-Pacific region. Seven of the CPGs were developed by retina specialists only, whereas two listed ophthalmologists more generally. PICOS registered on PROSPERO are broadly similar to those presented here; however, there are some data extraction criteria that were combined because of a lack of granularity among the retrieved CPGs.

**Table 1:** Characteristics of CPGs.

First author, year	Institution/professional group	Region /country	CPG development experts	Consensus method	Methods included literature review (Y/N)
Androudi 2016 [29]	N/R	Greece	Retina specialists	Consensus	N/R
Chaikitmongkol 2021 [22]	Asia-Pacific Vitreo-retina Society	Asia-Pacific	Retina specialists	Consensus	Y
Cheng 2022 [27]	N/R	Taiwan	Retina specialists	Anonymous voting	Y
Cruess 2012 [30]	Canadian Ophthalmological Society	Canada	Retina specialists	Consensus	Y
Flaxel 2020 [26]	American Academy of Ophthalmology	USA	Retina specialists	Consensus	Y
NICE 2018 [25]	National Institute for Health and Care Excellence	United Kingdom	Ophthalmologists, optometrists, general practitioners, community eye-health service managers, nurses, health economists, patients	Accordance with developing NICE guidelines manual	Y
Schmidt-Erfurth 2014 [28]	European Society of Retina Specialists	Europe	Retina specialists	Consensus	N/R
Tuuminen 2017 [24]	Finnish Ophthalmological Society	Finland	Ophthalmologists	Consensus	Y
Yeung 2021 [23]	Taiwan Retina Society	Taiwan	Retina specialists	Anonymous voting	N/R

*CPG* clinical practice guideline, *NICE* National Institute for Health and Care Excellence, *N/R* not reported, *Y/N* yes/no.

### Quality appraisal of CPGs using AGREE II

**Fig. 2:**
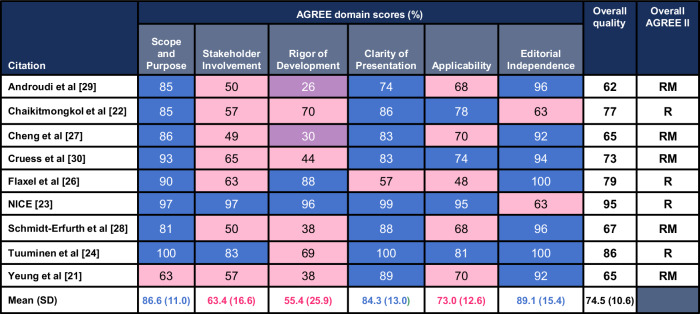
Standardised scores for each domain using the AGREE II instrument. Domain scores were rounded. Blue = high quality (>71%); pink = moderate quality (31–70%); purple = low quality (<30%). AGREE II, Appraisal of Guidelines for Research and Evaluation II; R, recommend; SD, standard deviation; RM, recommend with modifications.

Figure 2 summarises our findings after using the AGREE II appraisal instrument to analyse the quality of each CPG. Overall quality score of the CPGs, assessed by AGREE II, ranged from 62% to 95%, with the mean (standard deviation [SD]) being 74.5% (10.6). All CPGs were either “Recommended” or “Recommended with Modifications” by the reviewers. According to the domains of the AGREE II appraisal instrument, domain 1 (Scope and Purpose), domain 4 (Clarity of Presentation), and domain 6 (Editorial Independence) had the highest scores, with overall means (SD) of 86.6 (11.0), 84.3 (13.0), and 89.1 (15.4), respectively. The CPGs clearly described the overall objectives, health questions, and the population to whom the guideline is meant to apply. The different options for management of the condition or health issue were also clearly presented. Additionally, the recommendations were easily identifiable without ambiguity in language, and the views of the funding body did not influence the content of the CPG, with competing interests of guideline development group members addressed.

Out of the six domains, domain 3 (Rigor of Development) scored the lowest with the overall mean of 55.4 (25.9) followed by domain 2 (Stakeholder Involvement) 63.4 (16.6), and domain 5 (Applicability) 73.0 (12.6). Rigor of Development, which is scored based on the methodology followed during the guideline development, had the largest variety of scores among the CPGs. Furthermore, when evaluating multiple studies to make recommendations, meta-analyses were generally not conducted to combine the results of study outcomes which the reviewers accounted for in the scores determined for the criteria. Application of the CPG was clearly presented in four publications [22–25], which included elements such as use resource-constrained systems, flow charts to guide practice, etc. Furthermore, only 2/9 CPGs included a patient perspective [24,25].

In domain 2 (Stakeholder Involvement), the best-ranked CPG (NICE 2018 [25]) included a multidisciplinary approach, involving various professionals such as ophthalmologists, optometrists, general practitioners, community eye-health service managers, nurses, health economists, and patients. Additionally, in domain 5 (Applicability), the highest-scoring CPG included a thorough consideration of health economic evidence, which was mentioned at the end of every section. It also addressed crucial factors, such as barriers that need to be considered when evaluating the uptake of treatment for patients with AMD.

### Strength of recommendations on disease management

**Fig. 3:**
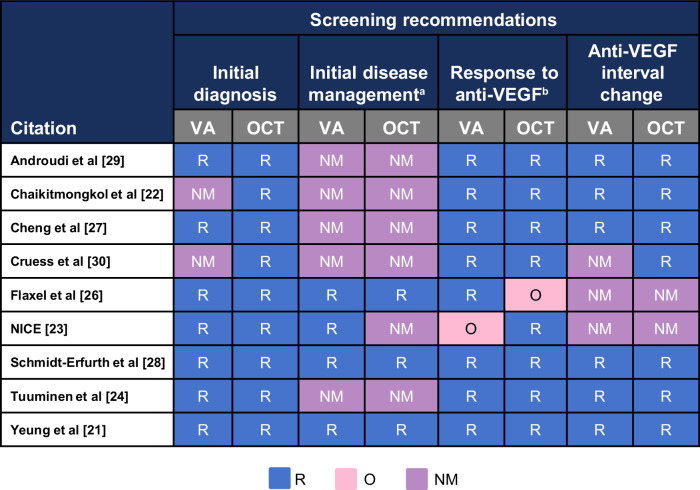
Screening recommendations for managing nAMD anti-VEGF therapy. ^a^ Management of initial pharmacological intervention. ^b^ Screening to assess disease progression. nAMD neovascular age-related macular degeneration, NM not mentioned, O optional, OCT optical coherence tomography, R recommend, VA visual acuity, VEGF vascular endothelial growth factor.

For initial diagnosis of AMD, all CPGs (*N* = 9) recommended OCT and seven recommended VA (Fig. 3). In terms of initial disease management of nAMD, two CPGs did not provide specific guidance regarding the management of different pharmacologic interventions.

Instead, these two CPGs focused specifically on recommendations for the treat-and-extend regimen, thus omitting any mention of managing various pharmacological interventions.

Recommendations on screening response to anti-VEGF were mentioned in all CPGs. Nearly all CPGs (*n* = 8/9) recommended using both VA or OCT to screen for anti-VEGF response to assess the progression of nAMD. Two CPGs recommended either VA or OCT to screen for anti-VEGF response to assess the progression of nAMD and suggested another screening method as optional [25,26]. The CPG with the highest overall AGREE II score recommends VA as optional when screening anti-VEGF response [25]. Conversely, the CPG with the second highest overall AGREE II score recommends OCT as optional when screening anti-VEGF therapy response [26]. Among the CPGs, other measures recommended to screen for anti-VEGF response include fluorescein angiography, biomicroscopy, colour fundus examination, and indocyanine green angiography. Six CPGs recommend screening for VA [22–24,27–29], and seven CPGs recommend screening for OCT when changing the anti-VEGF treatment interval [22–24,27–30]. Lastly, since steroids are not recommended for nAMD, recommendations on screening response to steroids were not mentioned in any of the included CPGs. Only one of the CPGs did not mention any recommendations regarding changing the treatment interval based on different screening measures.

## Discussion

This study evaluated CPGs for nAMD management and treatment, including understanding screening modalities on which to base prescribing of anti-VEGF agents. Eligible CPGs were published between 2012 and 2022, indicating that despite the evolving treatment landscape of nAMD since 2021, multiple CPGs have not yet been updated.

Unlike CPGs in other therapeutic areas (e.g., the National Comprehensive Cancer Network, the American College of Cardiology, the American Association for the Study of Liver Diseases), ophthalmology CPGs avoided being overly prescriptive with ophthalmologists and retina specialists, especially regarding treatment recommendations and guidelines to base management decisions. Our analysis of CPGs highlights the nearly universal recommendation to use OCT throughout the nAMD care continuum. The correlation between anatomical response and VA is evident in a variety of anti-VEGF clinical trials as well as real-world case studies and post-hoc analyses. Specifically, in nAMD, fluid increases may be a prognostic indicator of future vision decline [31]. Post-hoc analyses of the HAWK and HARRIER clinical trials demonstrated that reductions in fluid, regardless of type, early after the loading phase of an anti-VEGF agent (brolucizumab or aflibercept) is associated with positive VA outcomes [32]. Furthermore, recent research suggests that fluctuations in central subfoveal thickness correlate to increases in fibrosis, geographic atrophy, and macular fluid, which in turn leads to reductions in VA [33]. Although the use of OCT to measure disease activity is established in clinical practice, discordance between clinical trial disease assessment criteria and real world practice creates a degree of uncertainty around the effectiveness of anti-VEGF agents in the real world [18,19]. A lack of up-to-date CPGs may result in further variability in clinical practice.

Moreover, due to the rapid, progressive vision loss associated with the natural history of nAMD, there are more recent literatures investigating the long-term outcomes of the therapeutic interventions and also the sustainability and durability of treatment responses [34–37]. It is important for CPGs to incorporate the long-term outcomes of therapeutic interventions, as this highlights the public health necessity of providing treatment to those in need as early as possible, as well as ensuring sustainable therapy that warrants adherence and persistence. Therefore, societies and agencies should consider updating CPGs at regular intervals to ensure the most current recommendations are documented and give external stakeholders the broadest reach.

Across the six quality domains of the AGREE II criteria (Scope and Purpose, Stakeholder Involvement, Rigor of Development, Clarity of Presentation, Applicability, and Editorial Independence), it was observed that the domains of Rigor of Development, Stakeholder Involvement, and Applicability scored the lowest. Many of the CPGs did not state that SLRs were conducted during the development process. In addition, strengths and limitations of evidence described or health benefits, side effects, and risks were not reported consistently.

Specifically, the use of SLRs to thoroughly identify relevant clinical information ensures that CPGs are developed based on the leveraging of data versus relying on consensus among a limited number of providers to determine best practices [38]. The CPG [25] that scored the highest in the Rigor of Development domain of AGREE II also achieved the highest overall quality assessment score.

Notably, only 2/9 CPGs [24,25] included a patient perspective in their recommendations, with the overall score for CPGs being low for the Stakeholder Involvement domain. This an important limitation for existing CPGs in AMD, considering the chronic nature of the disease and high treatment burden experienced by patients [39]. In particular, patients undergoing treatments for nAMD often face treatment barriers such as time and travel pressures, caregiver dependency, and anxiety about intravitreal injections, which can increase the risk of undertreatment and nonadherence [40–43]. Therefore, it is crucial to incorporate patient perspectives into CPG recommendations to optimize the clinical efficacy of treatments by ensuring adherence and persistence [44]. To better incorporate patient perspectives into CPGs recommendations, while comparing treatments, it would be essential to conduct appropriate quality of life assessments (e.g. National Eye Institute Visual Function Questionnaire-25) [45] and treatment satisfaction questionnaires (e.g. Macular Disease Treatment Satisfaction Questionnaire, Port Delivery System Patient Preference Questionnaire) [46,47]. Additionally, due to the aging population in nAMD, it is inevitable that these population have other systemic health variables or comorbid conditions [48]. With a recent literature suggesting that systemic health variables such as a serum metabolomics could impact patients with nAMD in response to treatments [49], similar to other elements of medicine, involving a multidisciplinary team and considering patient’s comorbid conditions will provides a comprehensive perspective and expertise in addressing the diverse aspects of managing nAMD effectively.

In addition to healthcare providers, CPGs are often leveraged by regulatory and insurance policymakers to inform access strategies for implementing recommendations and resource allocation. Similar to above, the lack of an authoritative positions on treatment recommendations within ophthalmology CPGs and their variability present a potential challenge for reimbursement. Additionally, most of the CPGs assessed failed to address issues of applicability and resource constraints, which are key barriers to optimally implementing CPGs [50]. In the absence of CPGs, formulary management decisions may be more heavily influenced by policymaker budgets than clinical best practice.

The NICE CPG [25] had the highest overall quality AGREE II score, as it successfully incorporated the criteria in most of the AGREE II domains. The NICE CPG could serve as a prime example of how future CPGs should be developed. The NICE CPG not only included the views and preferences of the patients, but the systematic methods to search for evidence along with clearly defined criteria for evidence selection used in its development.

The methods for formulating the recommendations were clearly described in the CPG along with the strengths and limitations of the evidence. Lastly, the NICE CPG described facilitators and barriers to its application along with the consideration of the potential resource implications of applying the recommendations. All these elements highlight areas where future CPGs should focus on to develop more rigorous and comprehensive guidelines. By incorporating these elements, the CPG enhances the practical relevance and implementation of the recommendations, ultimately improving patient outcomes and healthcare delivery in the management of AMD inclusive of nAMD.

The limitations of this study were mostly because of the nature of SLRs, which are susceptible to publication bias. Publication bias occurs when a study’s findings affect its likelihood of publication (i.e., if editors, reviewers, or colleagues in the field are not interested in the information presented), in this case where clinicians may expect that OCT measurement is widely used in clinical practice. Despite this we were able to identify some variabilities, highlighting the need for rigorously developed, regularly updated CPGs. The included studies are also limited by the date range used of when the studies were first conducted, thus not including CPGs that subsequently became available. Furthermore, the search was limited to English-language publications, which could overlook relevant CPGs published in other languages. The AGREE II tool does not set a threshold for quality; therefore, the reviewers implemented a threshold of <30% as low quality and >70% as high quality across the individual domains. Additionally, the overall quality and recommendations for use in practice (Recommend, Recommend with Modifications, Do Not Recommend) were based on the reviewers’ judgement and could be considered subjective to the reviewers’ assessment of each CPG. However, the overall recommendation was made taking into consideration the AGREE II assessment process, which averaged the scores across multiple reviewers to minimise bias. Moreover, some of the domain items may not have been considered in scope or budget for professional groups that developed the CPGs (e.g., methods for updating the guidelines or establishing monitoring/auditing criteria). Lastly, our abstraction focused on pharmacological interventions, particularly anti-VEGF agents, which may have omitted alternative treatments, such as laser (photocoagulation) and intraocular steroids that could rarely be preferred depending on the clinical settings.

In summary, this study contributes novel insights into the landscape of CPGs for nAMD by performing a thorough, geographically inclusive systematic review and assessment using the AGREE II instrument. By identifying and evaluating CPGs from a wide geographical distribution, a significant variation in methodological rigor and stakeholder involvement across different regions has been highlighted-gaps such as the limited inclusion of patient perspectives and economic considerations in CPG development. Although the extensive use of OCT and VA as diagnostic and monitoring tools has been widely recognized, the heterogeneity in recommendations regarding their application still exists. By providing a comprehensive appraisal and identifying areas for improvement, the study sets a foundation for the development of more robust, inclusive, and stakeholder-engaged CPGs that cater to various healthcare settings, potentially enhancing the management and treatment outcomes for nAMD patients.

## Summary

### What was known before

- The majority of nAMD randomised controlled trials assessing anti-VEGF agents historically used VA as the primary endpoint, but published research has shown that there may be limited correlation between optical coherence tomography (OCT) and VA. It is important to improve our understanding of how retina specialists interpret OCT and VA and how they are used in clinical practice. As such, obtaining a comprehensive understanding of how explicitly the CPGs support the use of these assessments in the care continuum would be helpful to minimise the inequality of patient care.

### What this study adds

- Our analysis of CPGs highlights the nearly universal recommendation to use OCT throughout the nAMD care continuum. Although the use of OCT is established in clinical practice, lack of up-to-date CPGs may still result in variability in clinical practice. Therefore, societies and agencies should consider updating CPGs at regular intervals to ensure the most current recommendations are documented and give external stakeholders the broadest reach. Current nAMD CPGs are robust in their scope and purpose, clarity of presentation, and editorial independence. However, more rigorous methodological development and broader stakeholder involvement from patients and caregivers could improve the trustworthiness and transparency of future CPGs for nAMD disease management.

## Supplementary information

Supplementary Table 1

Supplementary Table 2

## Data Availability

The datasets generated during and/or analysed during the current study are available from the corresponding author on reasonable request.
