# Supplementary material for: Systematic review of clinical practice guidelines for the management of neovascular age-related macular degeneration
Source: Eye (Lond). 2025 May 15;39(11):2223–30. doi: 10.1038/s41433-025-03829-8 (PMC12274593; doi:10.1038/s41433-025-03829-8)
Supplement: Supplementary file 1 — Supplementary Table 1 [file 41433_2025_3829_MOESM1_ESM.docx]

**Supplementary Table S1.** Detailed search strategy.

| **#** | **Search terms (title/abstract screen)** | **Hits** |
| --- | --- | --- |
| 1 | “neovascular age-related macular degeneration” OR “wet age-related macular degeneration” OR “neovascular age related macular degeneration” OR “diabetic macular edema” OR “diabetic macular oedema” OR “diabetic retinopathy” OR “retinal vein occlusion” | 88 101* |
| 2 | Guideline* OR “practice guidelines” OR (“practice” AND “guidelines”) OR (“best” AND “practice”) OR “best practice” OR “Preferred Practice Pattern” OR consensus OR “executive summary” OR (executive AND summary) OR “Expert consensus” OR “Scientific Statement” OR “consensus paper” OR “consensus statement” | 298 866 |
| 3 | #1 AND #2 | 163 |
| 4 | #3; Remove Duplicates; Publication after 2010 | 133 |
| 5 | Grey literature and backwards citation tracking | 14 |

*Search for additional ophthalmologic conditions conducted in tandem (i.e., retinal vein occlusion, neovascular age-related macular degeneration).
