# Supplementary material for: Systematic review of clinical practice guidelines for the management of neovascular age-related macular degeneration
Source: Eye (Lond). 2025 May 15;39(11):2223–30. doi: 10.1038/s41433-025-03829-8 (PMC12274593; doi:10.1038/s41433-025-03829-8)
Supplement: Supplementary file 2 — Supplementary Table 2 [file 41433_2025_3829_MOESM2_ESM.docx]

**Supplementary Table S2.** AGREE II quality instrument domains [10]

| **AGREE II domain** | **Criteria evaluated** |
| --- | --- |
| 1. Scope and Purpose | • The overall objectives of the guideline are specifically described  • The health questions covered by the guideline are specifically described  • The population to whom the guideline is meant to apply is specifically described |
| 2. Stakeholder Involvement | • The guideline development group includes individuals from all relevant professional groups  • The views and preferences of the target population have been sought  • The target users of the guideline are clearly defined |
| 3. Rigor of Development | • Systematic methods were used to search for evidence  • The criteria for selecting the evidence are clearly described  • The strengths and limitations of the body of evidence are clearly described  • The methods for formulating the recommendations are clearly described  • The health benefits, side effects, and risks have been considered in formulating the recommendations  • There is an explicit link between the recommendations and the supporting evidence  • The guideline has been externally reviewed by experts before publication  • A procedure for updating the guideline is provided |
| 4. Clarity of Presentation | • The recommendations are specific and unambiguous  • The different options for management of the condition or health issue are clearly presented  • Key recommendations are easily identifiable |
| 5. Applicability | • The guideline describes facilitators and barriers to its application  • The guideline provides advice and/or tools on how the recommendations can be put into practice  • The potential resource implications of applying the recommendations have been considered  • The guideline presents monitoring and/or auditing criteria |
| 6. Editorial Independence | • The views of the funding body have not influenced the content of the guideline  • Competing interests of guideline development group members have been recorded and addressed |
| AGREE II, Appraisal of Guidelines for Research and Evaluation II. | |
